# The Application of Small-Angle Light Scattering for Rheo-Optical Characterization of Chitosan Colloidal Solutions

**DOI:** 10.3390/polym10040431

**Published:** 2018-04-13

**Authors:** Piotr Owczarz, Patryk Ziółkowski, Marek Dziubiński

**Affiliations:** Department of Chemical Engineering, Lodz University of Technology, 90-924 Lodz, Poland; piotr.owczarz@p.lodz.pl (P.O.); marek.dziubinski@p.lodz.pl (M.D.)

**Keywords:** chitosan, rheology, light scattering, SALS

## Abstract

In the recent studies on chitosan hydrogels, it was found that understanding both rheological and structural properties plays an important role in their application. Therefore, a combination of two independent techniques was applied to investigate micro- and macroscopic properties of chitosan colloidal system. Studies on viscous properties, as well as the sol-gel phase transition process, were performed using rheological methods coupled with the small angle light scattering (SALS) technique. Based on the anisotropy of scattering patterns obtained during rotational shear tests, it was found that the chitosan solution reveals two different behaviors delimited by the critical value of the shear rate. Below a critical value, chitosan clusters are deformed without breaking up aggregates, whereas after exceeding a critical value, chitosan clusters apart from deformation also breakup into smaller aggregates. The values of the radius of gyration determined by applying the Debye function allow one to state that with an increase of chitosan concentration, molecule size decreases. An analysis of the light scattering data from the temperature ramp test showed that with an increase of temperature, the level of polymer coil swelling increases. Simultaneously, the supply of thermal energy leads to a neutralization of the charge of chitosan chains. As a consequence, the formation of intermolecular links occurs and a gel structure is formed.

## 1. Introduction

Hydrogels are an interesting group of materials commonly used in medicine and pharmacology [1,2]. This is due to the biophysical similarity of hydrogels to soft tissues, as well as their responsiveness to various environmental changes. The most commonly used form of hydrogel is three-dimensional matrices that received found wide-spread application in the life sciences, including tissue engineering and smart drug delivery systems [3,4,5].

In manufacturing of hydrogel matrices for medical applications, biocompatible and biodegradable natural polymers are sought for. One such compound is chitosan, a polysaccharide obtained by deacetylation of chitin, which is a natural building component of crustaceans. A suitable buffering and cross-linking agent (e.g., disodium β-glycerophosphate) is used to prepare colloidal chitosan solutions that undergo phase transition under the influence of temperature increase in the physiological pH range [6,7]. In such systems, the main interactions responsible for the sol-gel phase transition are physical intermolecular forces such as hydrophobic interactions among chitosan chains. These interactions are a result of the neutralization of amino groups triggered by external stimuli such as temperature increase [8,9].

In the recent studies on chitosan hydrogels, it was found that understanding both macroscopic properties related to rheological characteristics of the systems and microscopic properties characterized by the size, shape, and chitosan molecule conformation play an important role in their application. The recent literature presents many studies depicting the influence of external stimuli (temperature, concentration, pH) and internal stimuli (e.g., molecular weight, degree of deacetylation) on rheological properties of colloidal chitosan solutions [7,10,11,12]. However, there are few studies analyzing both the rheological and microscopic properties of these media and a relationship between them.

One of the most frequently used techniques to describe the conformation of chitosan molecules is the Mark-Houwink equation [*η*] *= KM^a^*, in which [*η*] is the intrinsic viscosity, *M* is the molecular weight, and *K* and *a* are the Mark-Houwink constant and the exponent, respectively. The type of solvent used and associated differences in the ionic strength and pH of the solutions produce a different conformation of polysaccharide molecules in the solution [13,14,15]. Depending on the value of the exponent *a*—determined experimentally for standard solutions of known molecular weight—it was found that chitosan molecules in colloidal solutions assume the form of hard spheres and collapsed coils (*a* < 0.5), flexible and random coils (0.5 < *a* < 1.2), and disentangled rigid or rod-like configurations (*a* > 1.2).

The emergence of new techniques taking advantage of the analysis of scattering of a polarized, monochromatic beam of light allowed one to obtain more accurate and precise information on the microscopic properties of the materials tested. Among these techniques, a dynamic and static light scattering can be distinguished (DLS and SLS) [16]. In particular, significant progress has been made in the development of small angle light scattering (SALS). These techniques are used to study structures on a microscale. Their undoubted advantage is also the possibility of non-destructive testing of the phase transition or phase separation phenomena. Light scattering techniques provide valuable information about the sizes of polymer structures studied, such as radius of gyration *R_g_* or hydrodynamic radius *R_h_*, for instance. They also allow one to determine the conformation of molecules and follow the time evolution of these changes.

Over the last few years, several experimental works presenting the implementation of the SALS technique in measuring systems have been published [17,18,19,20,21,22,23]. In these works, the measurements of media properties were performed with the use of the Anton Paar MCR 502 rotational rheometer coupled with SALS optical system [22,23]. The device enables a simultaneous registration of rheological and microscopic properties of the medium. The dependence of the macroscopic, mechanical properties of the medium on a microstructure formed by the molecules of the tested material allows for a more comprehensive investigation of the mechanisms responsible for intermolecular interactions that occur in the tested material during mechanical deformation, phase transition processes, etc.

In this work, we aimed to study the microstructural and macroscopic properties of chitosan colloidal systems. A comprehensive analysis of properties of colloidal chitosan solutions using rheological methods combined with a simultaneous SALS measurements was conducted in the study. The effect of shear deformation on the structural organization of the chitosan molecules was carried out by analyzing the anisotropy of the obtained scattering patterns. The non-isothermal sol-gel phase transition process was also performed, taking into account different concentrations of colloidal chitosan solutions. To gain further insights into the gelation process, microstructural properties besides rheological behavior were studied based on light scattering data.

## 2. Materials and Methods

### 2.1. Materials and Solution Preparation

The chitosan obtained from crab shells, supplied by Sigma-Aldrich (Sigma-Aldrich Sp. z o. o., Poznan, Poland) was used for the study (product no. 50494, lot no. 0001424218). The weight average molecular mass M_w_, determined by gel permeation chromatography (GPC/SEC), was 680 kg∙mol^−1^. The degree of acetylation (DA), determined by the titrimetric method, was 18.2%. A cross-linking agent was disodium β-glycerophosphate, while 0.1 M aqueous hydrochloric acid was used as a solvent.

Chitosan solutions were prepared by dissolving four different amounts of chitosan, each in 8 g of aqueous hydrochloric acid solution. Concentrations expressed as the ratio of chitosan mass to solvent mass yield the values of 2.5, 3, 3.5, and 5% (*w*/*w*). After dissolution, the vessel containing the sample was covered and left for 24 h at room temperature; after about 3 h, the sample was again stirred to achieve better dispersion of polymer. After 24 h, the solution was placed at 5 °C for 2 h. After this time, 2 g of cooled and previously prepared solution of disodium β-glycerophosphate was added gradually to the chitosan solution and stirred at the same time. The solution of the cross-linking agent was obtained by mixing distilled water and disodium β-glycerophosphate at a 1:1 ratio.

### 2.2. Rheological Experiments

The measurements were carried out using the Anton Paar Physica MCR 502 rheometer (Anton Paar, Warszawa, Poland) equipped with an integrated SALS optical analysis system. The schematic presentation of Rheo-SALS system has been provided in the Supplementary Materials (Figure S1). The measurement system of the rheometer used in rotational tests was a double-gap system. To determine viscosity of the medium, the sample was subjected to shearing for 60 s at subsequent fixed shear rates of 1, 5, 10, 20, 50, 100, 200, and 500 s^−1^, repeating the procedure toward decreasing shear rate values. The process of thermo-induced gelation was carried out in a plate-plate system under oscillatory shear at fixed strain amplitude *γ* = 1%, angular frequency *ω* = 5 rad/s, and gap size of 0.3 mm. The samples were heated from 5 °C (storage temperature) to 80 °C, maintaining constant heating rate of 1 K/min.

### 2.3. Small Angle Light Scattering Experiments

Experiments recording light scattering data with simultaneous measurement of rheological properties were carried out using specially designed glass parts of the measurement system—double gap DG32-21/AL/CX and plate-plate PP43/GL-HT. The schematic presentation of measurement systems has been provided in the Supplementary Materials (Figure S2). The light source was 10 mW LED diode laser operating at a wavelength of 658 nm. In addition, the system contained a polarizer located in front of the laser and an analyzer placed below the sample, allowing two types of measurements: polarized (polarizer and analyzer parallel) and depolarized (polarizer and analyzer perpendicular). In the present study, the measurements were carried out only in the polarized configuration. Once the laser beam has passed through the sample, the main light beam was stopped at the beam-stop contained in the lens system, while scattered light was collected on the screen. The distance between the screen and the sample was 17.15 cm. The resulting two-dimensional scattering pattern was captured by a CCD camera. The RheoCompass^TM^ software (Anton Paar GmbH, Graz, Austria) provided by the manufacturer enable to real-time registration of scattering pattern. The obtained scattering patterns were analyzed using the NewSALS software (version 2.02) developed by the Laboratory of Applied Rheology and Polymer Processing (K.U. Leuven, Belgium) to determine the dependence of intensity *I* as a function of scattering vector *q*. The analyzed scattering vector *q* was approximately in the range 0.1–4.2 µm^−1^. The scattering vector was defined as *q* = (*4π/λ*)*sin*(*θ*/*2*), in which *λ* was the wavelength of the incident laser beam and *θ* was the scattering angle.

## 3. Results and Discussion

### 3.1. The Effect of Shear Rate on Structural and Viscous Properties

**Figure 1:**
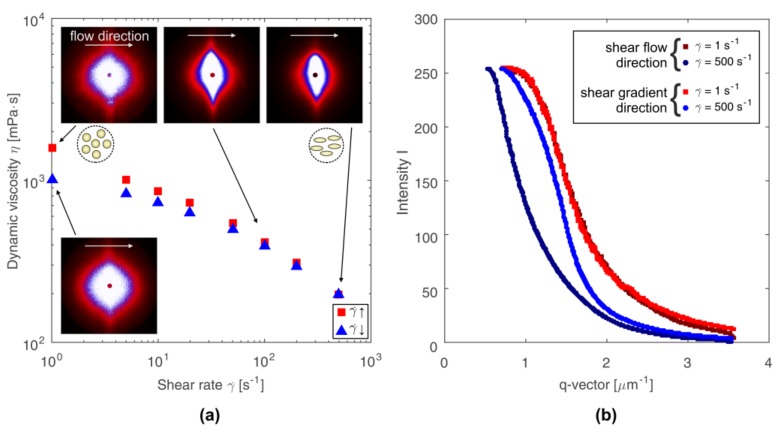
(**a**) Viscosity curves for 2.5% (*w*/*w*) chitosan solution determined for increasing (γ̇↑) and decreasing (γ̇↓) shear rate with scattering patterns obtained at 40 °C. (**b**) The q-vector dependency of the scattering intensities horizontally across (shear flow direction) and vertically across (shear gradient direction).

Viscosity curves for the 2.5% (*w*/*w*) chitosan solution, determined using the double gap geometry, along with scattering patterns and q-vector dependency of the scattering intensities in mutually orthogonal directions, are shown in Figure 1. Viscosity of the tested solution decreases with the increase of the shear rate, showing the features of a shear-thinning, non-Newtonian fluid. Simultaneously, there is hysteresis between the curves obtained for increasing and decreasing shear rate, which indicates thixotropic properties of the tested solution. Observation of the shear thinning behavior in the entire considered shear rate range suggests that in this range the colloidal systems are characterized by fast relaxation processes. The progressive decrease in the viscosity of the chitosan solution with the shear rate increase indicates a gradual breakup of intermolecular links.

Scattering patterns presented in Figure 1a show the distribution of scattering intensity on the plane of scattering vector *q*, for the initial (*γ̇* = 1 s^−1^), intermediate (*γ̇* = 100 s^−1^), and final (*γ̇* = 500 s^−1^) states during the measurement at increasing shear rate. Figure 1a also shows the scattering pattern obtained at shear rate of 1 s^−1^ during the test of decreasing shear rate. The differences in the scattering patterns obtained for initial and final state result from the thixotropic properties of the medium. It is possible to observe a change in the shape of the scattering pattern from circular to elliptical with increasing shear rate and a simultaneous decrease in viscosity. This change represents orientations and deformations of the polymer domains [24,25,26].

**Figure 2:**
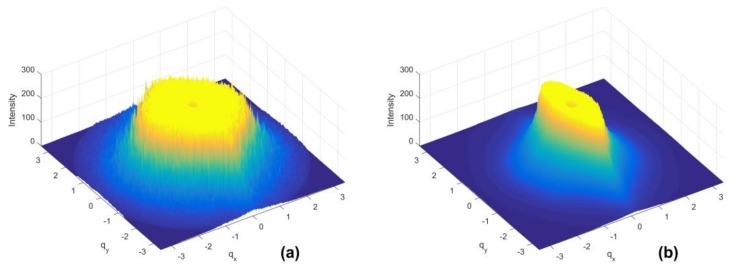
Three-dimensional distribution of light intensity on the scattering vector plane for 2.5% (*w*/*w*) chitosan solution at 40 °C. (**a**) Data for shear rate 1 s^−1^. (**b**) Data for shear rate 500 s^−1^.

For low shear rate values, slight differences were observed between intensity distribution along the mutually perpendicular directions *q_x_* and *q_y_*, which indicates a weak deformation of the molecules (see the intensity curves for lowest shear rate on Figure 1b). The higher the shear rate, the more deformed and oriented the molecules are, leading to a more stretched scattering pattern. Anisotropy of the scattering patterns, which increases as the shear rate increases, reflects the influence of shear forces on the deformation of the ordered structure of polymer chains in the solution. The scattering pattern, which is elliptically shaped and oriented perpendicular to the shear flow direction, results from the orientation and stretching of molecules along the shear flow direction. Intensity distribution on the scattering vector plane presented in a three-dimensional coordinate system is shown in Figure 2.

In the wave vector region covered in these measurements, the power law (*I(q)~q^-n^*) can describe the *q* dependence of the light intensity. In the wave vector regime *qR_g_ > 1*, the experiment probes the cluster fractal dimension, and the value of the power exponent *n* provides structural information on the fractal dimension of molecules [27,28]. Based on data of the scattering pattern at 20 °C for the lowest shear rate, the value of exponent *n* was approximately ~2.7. This value indicates that the polymer chains have an intermediate conformation between a collapsed coil (*n* = 3) and swollen Gaussian coil (*n* = 2). Molecules formed in this manner are deformed by shear forces, and the resulting scattering pattern reveals more prominent anisotropy for higher shear rate values (Figure 2b).

### 3.2. Quantification of Scattering Pattern Anisotropy

Based on the three-dimensional scattering patterns presented in Figure 2, it is possible to quantify anisotropy. Anisotropy *ϵ* is determined based on the eigenvalues of the second-order tensor of the intensity distribution [29,30,31]:

(1) $$\epsilon =\frac{\sqrt{{\left(M_{xx}-M_{yy}\right)}^{2}+4\tau_{xy}^{2}}}{S_{I}}$$

(2) $$M_{xx}=\int^{\text{​}}q_{x}q_{x}I\left(q,\dot{\gamma },t\right)dq$$

(3) $$M_{yy}=\int^{\text{​}}q_{y}q_{y}I\left(q,\dot{\gamma },t\right)dq$$

(4) $$\tau_{xy}=\int^{\text{​}}q_{x}q_{y}I\left(q,\dot{\gamma },t\right)dq$$

(5) $$S_{I}=\int^{\text{​}}I\left(q,\dot{\gamma },t\right)dq$$

in which *M_xx_, M_yy_,* and *τ_xy_* are the second-order intensity tensors, and *S_I_* is the summary intensity.

**Figure 3:**
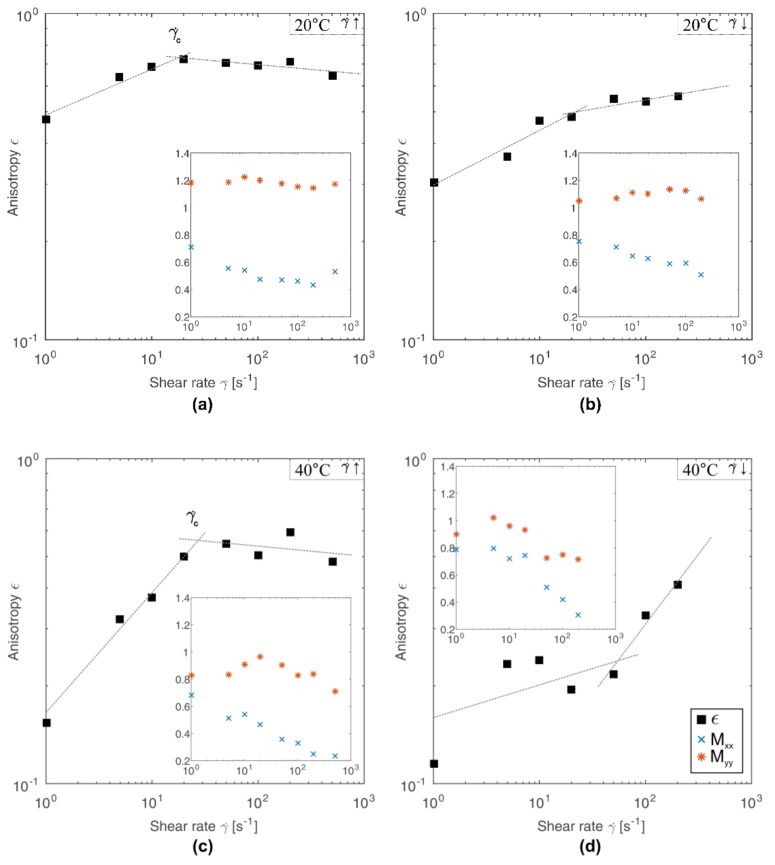
Changes in anisotropy ϵ as a function of shear rate. The inner plot show changes in the second-order tensors M_xx_ and M_yy_ as a shear rate function. Measurements were made for increasing (γ̇↑) and decreasing (γ̇↓) shear rates.

The dependence of anisotropy on the shear rate is shown in Figure 3. With the increase of shear rate in the first stage of the experiment, an increase of anisotropy is observed, which indicates progressive deformation of the molecules. After reaching a certain critical shear rate value *γ̇_c_*, anisotropy reaches its maximum value. Once the maximum of anisotropy is exceeded, any further increase of shear rate no longer causes significant changes in anisotropy, and a minimal decrease of this parameter is observed. For chitosan solutions tested at both 20 °C and 40 °C (Figure 3a,c, respectively), the determined critical shear rate is approximately 20 s^−1^. Similar behavior is observed at decreasing shear rate (Figure 3b,d), although in this case the value of anisotropy decreases in the entire shear rate range. Additionally, near the value of critical shear rate the change of the slope of function describing the experimental data occurs.

The occurrence of a critical shear rate indicates the structural reorganization of molecules. Before reaching the critical point, the molecule deformation increases. The higher the shear force, the higher the orientation of the molecule along the flow direction. After exceeding the critical point, the deformation remains relatively constant despite the increase of shear forces. Simultaneously, however, the dynamic viscosity of the tested system decreases in the entire considered range of shear rates without revealing of a critical point. Lack of significant changes in anisotropy with a simultaneous decrease in viscosity may suggest that when the critical point is reached, chitosan aggregates break up. However, lack of significant changes in anisotropy may also result from excessive length-scale that can be covered by using SALS technique, whereas continuous decrease in viscosity is the result of orientation and ordering of chitosan chains along the flow direction.

The occurrence of chitosan chains in the form of Gaussian coils and acting on them with low shear forces leads to their deformation. However, associative forces merging the polymer coil are high enough to limit deformations only to stretching of chitosan clusters along the flow direction. In such conditions the aggregation processes can be accelerated, because the shear flow tends to bring molecules together in the form of aggregates and leads to their faster formation than Brownian motion does at quiescence state [32]. The situation changes the critical shear rate is exceeded. High shear forces are able to overcome the associative forces merging the chitosan cluster. In addition, high shear rates lead to mutual collisions between the polymer aggregates. As a result, chitosan clusters, apart from their shape deformation, also break up into smaller aggregates. Such behavior may explain the lack of significant changes in the anisotropy of scattering patterns above the critical shear rate (Figure 3), while the viscosity of the medium (Figure 1a) shows a continuous decrease in the entire range of shear rate.

### 3.3. The Sol-Gel Phase Transition and Temperature of the Gel Point

**Figure 4:**
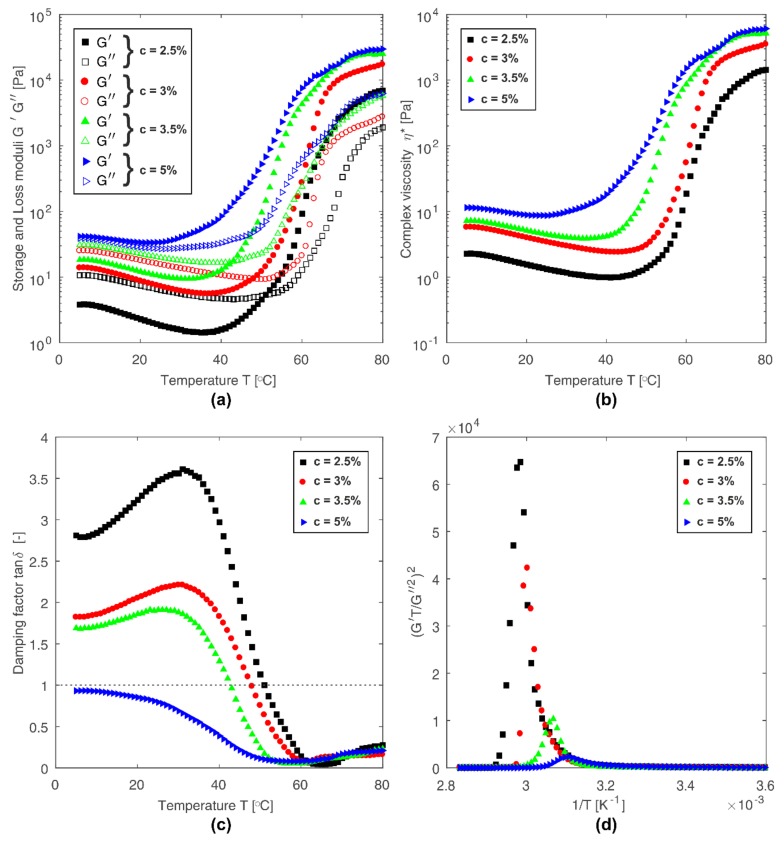
Rheological data of phase transition for all tested samples. (**a**) Changes in storage G’ and loss G” moduli as a function of temperature. (**b**) Complex viscosity η* as a function of temperature. (**c**) Damping factor tanδ as a function of temperature. (**d**) Gelation temperature determined by the Fredrickson-Larson method.

The process of sol-gel phase transition was investigated by two independent methods: rheological and SALS technique. The process of thermo-induced phase transition, tested by rheological techniques, was carried out in a plate-plate rheometer measuring system for samples with four different chitosan concentrations under oscillatory shear conditions. The temperature evolution of changes in storage G’ and loss G” moduli are illustrated in Figure 4a. All tested samples followed the characteristic variation of dynamic moduli for the sol-gel phase transition. Curves presented in Figure 4a indicate also the effect of chitosan concentration on the gelation process. With increasing concentration, the system reveals higher values of G’ and G” moduli. This is a result of stronger cross-linking forces between polymer chains for systems with higher concentrations. The predominance of elastic forces over viscous ones for more concentrated solutions is clearly visible in the diagram presenting damping factor *tanδ* as a function of temperature (Figure 4c). The concentration of the chitosan solution also has a significant effect on the phase transition temperature defined as a crossover point of the storage and loss moduli, i.e., for *tanδ* = G”/G’ = 1. As the concentration increases, the gelation temperature decreases. However, it should be noted that crossover point method does not specify the exact gelation point position but only gives an approximation where gelation is occurring.

An alternative approach to determine the gelation point based on rheological data is the method described by Fredrickson-Larson (F-L) [33,34]. According to the F-L theory, the ratio (G’T/G”^2^)^2^ as a function of inverse temperature 1/T demonstrates divergence behavior near the gelation point. The peak positions of the curves depicted in Figure 4d determine the gelation temperature. The determined temperatures of phase transition according to F-L theory reveal a decrease of gelation temperature with an increase of concentration of chitosan solutions. However, these values are slightly higher than those determined based on the crossover point of G’ and G” moduli (Figure 4c).

In order to determine the gelation temperature based on data obtained independently by the SALS method, the Ornstein-Zernike equation was used [35,36]:

(6) $$I\left(q\right)=\frac{I_{0}}{1+q^{2}\xi^{2}}$$

in which *I*_0_ is the intensity for *q* = 0, and *ξ* is the correlation length.

Parameters *I*_0_ and *ξ* can be determined from the y-intercept and the slope of the Zimm plot (1/*I*(*q*) vs. *q^2^*), respectively. With a set of intensity curves as a function of the scattering vector for the entire gelation process, the value of the intercept as a function of temperature can be determined. The change of the intercept from negative to positive value indicates the gelation point.

**Figure 5:**
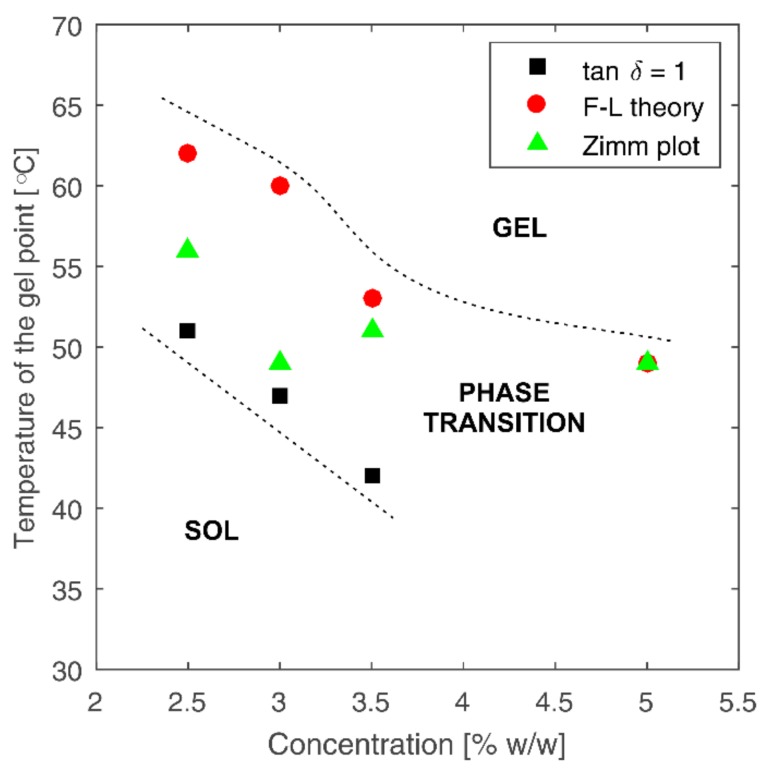
Gelation temperatures for all tested samples at different concentrations determined on the basis of rheological and light scattering data.

A comparison of phase transition temperatures determined by various methods for different chitosan concentrations is shown in Figure 5. Gelation temperatures determined by the crossover point of the curves of moduli G’ and G” (*tanδ* = 1) show the lowest values. For the 5% solution, it was not possible to determine the gelation point using this method, because the value of storage modulus G’ was higher than that of loss modulus G” (*tanδ* = G”/G’ < 1) in the entire temperature region covered in conducted measurements. Gelation points determined by other methods, i.e., the F-L theory and Zimm plot, yield similar values of gelation temperature for higher concentrations (3.5 and 5%). Based on the gelation temperatures presented in Figure 5, three characteristic phase areas for the investigated process can be distinguished. The initial sol phase is separated from the final gel phase by the phase transition area in which a rapid increase in the values of dynamic moduli of the tested medium occurs.

**Figure 6:**
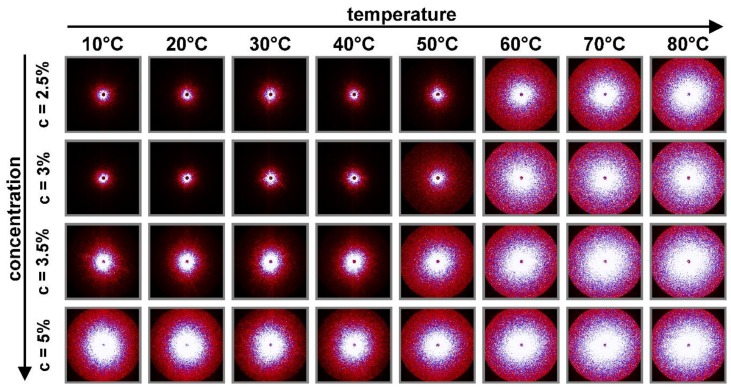
Comparison of the light scattering patterns obtained for different polymer concentrations and temperatures.

Simultaneous registration of light scattering data during rheological tests allows one to gain wider insight into phase transition process. Figure 6 shows the resulting light scattering patterns depending on temperature and concentration. As can be seen, the intensity increases with increasing temperature and concentration. This increase of intensity may result from increase in the number of scatters that depend on the concentration and temperature. Some of the researchers [32,37,38] investigating the phenomenon of phase transitions reported similar behavior of scattering pattern and explain it as a signature of formation of multi-chain associations.

**Figure 7:**
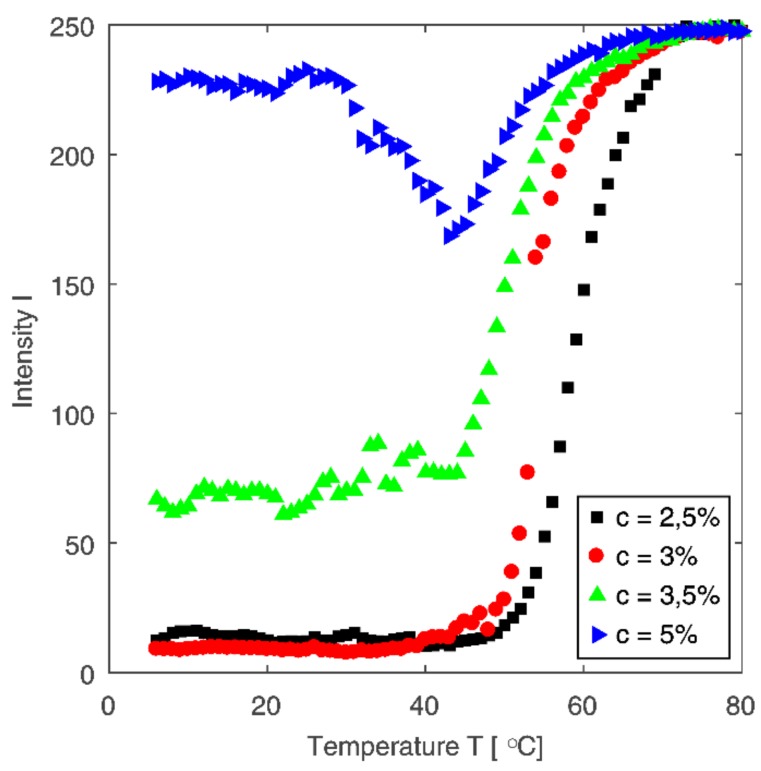
Dependence of intensity (at fixed value of scattering vector *q* = 1.5 μm) on temperature for all tested samples.

Figure 7 shows the temperature evolution of intensity for a fixed value of scattering vector (*q* = 1.5 μm) for four tested samples at different concentrations. The evolution of intensity curves shows similarity to rheological data (cf. Figure 4b). The higher the solution concentration, the higher the intensity in the initial phase of the process. The phase transition can be observed as a rapid increase in intensity. The shape of curves with distinctive inflection point shown in Figure 7 is similar to the shape of curves representing changes in complex viscosity as a function of temperature shown in Figure 4b. The exception is the intensity curve for the most concentrated chitosan solution, which, in contrast to the other tested solutions, shows predominance of elastic properties over viscous ones in the entire considered temperature range.

### 3.4. Radius of Gyration and Chitosan Chains Conformation

**Figure 8:**
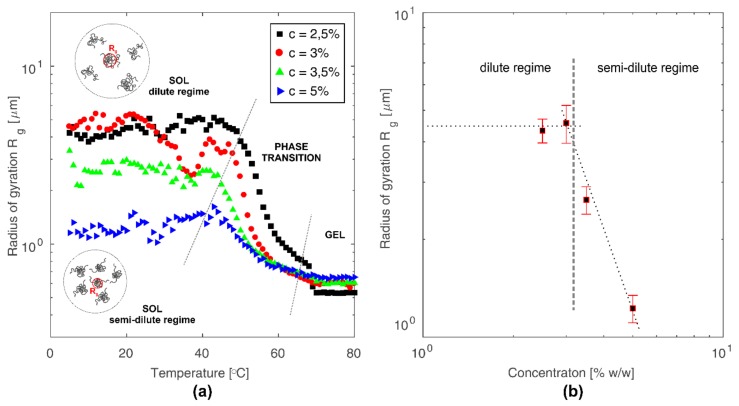
(**a**) Changes in the radius of gyration as a function of temperature for all tested concentrations of colloidal chitosan solutions. (**b**) Changes in the radius of gyration expressed as an average value of gyration radii at the initial phase of the process (sol) as a function of concentrations of colloidal chitosan solutions.

The radius of gyration of chitosan molecules in the colloidal solution was determined on the basis of the dependence of light intensity on the scattering vector. The radius of gyration determines molecule structure compactness [39]. The lower the value of radius of gyration, the tighter is packing of molecules, which in the case of linear polysaccharides means that polymer chains assume more collapsed form. The value of gyration radius for the tested colloidal solutions of chitosan was determined by fitting experimental data by Debye function [40,41]:

(7) $$I\left(q\right)=2\frac{I_{0}}{x^{2}}\left(e^{-x}-1+x\right)$$

in which *x = q*^2^*R_g_*^2^, and *R_g_*^2^ is the mean square radius of gyration. For the tested solutions at concentrations 2.5, 3, 3.5, and 5% (*w*/*w*), the values of gyration radius were to 4.33, 4.57, 2.65, and 1.23 μm, respectively. Changes in the radius of gyration as a function of temperature and concentration are presented in Figure 8.

**Figure 9:**
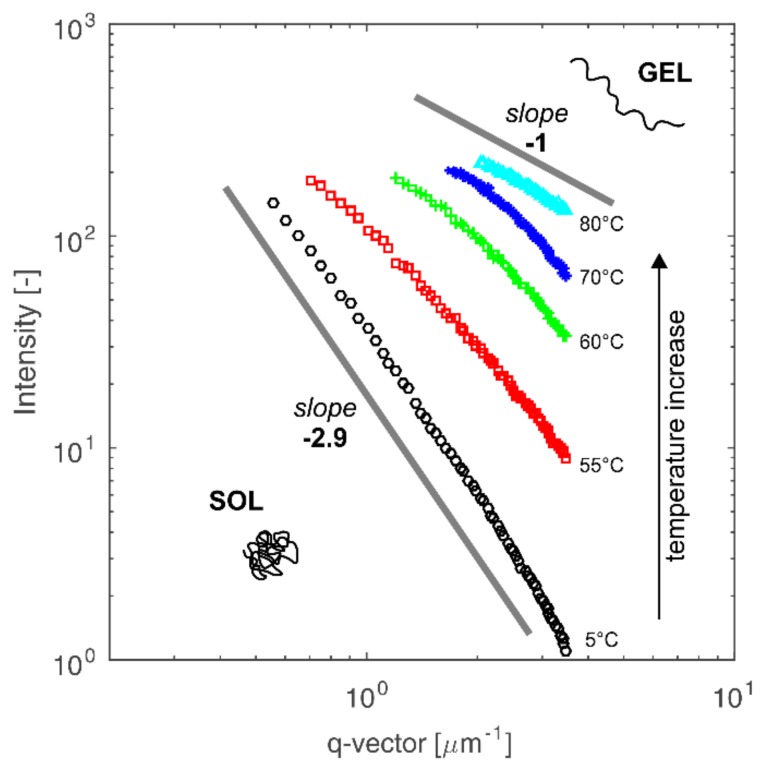
Dependence of intensity as a function of scattering vector in the log scale, enabling determination of exponent n. Data for the sample with concentration 2.5% (*w*/*w*).

In order to determine changes in the conformation of chitosan molecules during the gelation process, experimental data were approximated by the power-law dependence of the values of intensity on scattering vector *I(q)~q^-n^*. According to the Porod law, the value of exponent *n* is related to the volumetric (mass or porous) fractal dimension of the scattered molecules [27,42]. The value of the exponent *n* = 4 represents a smooth surface for the scattering particle and indicates that the system is close to macroscopic phase separation. In the case of polymers, parameter *n* is related to the type of conformation assumed by the polymer chains. For stiff, collapsed coils, parameter *n* assumes the value *n* = 3. For Gaussian coil, *n* is equal to 2. A further decrease of parameter *n* indicates the intensification of coil swelling and its disentangling to a rigid rod form. Changes in intensity as a function of scattering vector and the obtained values of curves slopes for the sample with 2.5% concentration are shown in Figure 9.

Analysis of the determined values of exponent *n* allows one to state that at low temperature in the sol phase, dispersed chitosan molecules are present in the form of Gaussian coils. Phenomenon of the protonation of amino groups in the chitosan molecule, allowing polymer dispersions in the continuous phase, also determines association effect in the polymer coil. With an increase of chitosan concentration, the number of amino groups capable of being protonated also increases, while the number of H^+^ ions derived from the solvent remains constant. Therefore, it can be assumed that with the increase in chitosan concentration, the average degree of protonation of a single polymer coil decreases. The lower charge of the chitosan chain leads to a more compact conformation resulting from the greater prevalence of the hydrophobic forces, determined by the neutral charge of the chitosan amino groups, over the hydrophilic forces, which are dependent on the positive charge of the chitosan amino groups. Therefore, in more concentrated solutions there are more tangled coils of smaller size but occurring in a larger number than in diluted solutions. Schematically, this phenomenon is presented in Figure 8a. The decrease in radius of gyration for colloidal chitosan solutions at a concentration above 3%, as shown in Figure 8b, is related to the transition from the dilute regime to semi-dilute regime [43].

Stability of the studied colloidal chitosan systems, resulting from the presence of hydrogen bonds among positively charged amino groups of chitosan and solvent molecules, changes under the influence of temperature increase. Energy supplied in the form of heat leads to the neutralization of charge of the chitosan chains [9,44]. As a result, the contribution of hydrophobic effect increases, which causes the precipitation and gelation of chitosan system. Simultaneously with temperature increases, the level of polymer coil swelling increases as indicated by the decrease in the exponent *n* presented in Figure 9. This leads to the formation of intermolecular links and gel structure.

## 4. Conclusions

The investigation of the viscous properties, as well as the sol-gel phase transition process, carried out using rheological methods coupled with the SALS technique provides a comprehensive survey of the colloidal chitosan solutions behavior. Such studies have shown that in colloidal chitosan solutions, the polymer domains occur in the form of Gaussian coils. Based on rotational shear measurements, it was found that chitosan solution reveals two different behaviors delimited by the critical value of the shear rate. Below the critical value, shear forces lead to deformation of chitosan clusters without breaking up aggregates. In such conditions, the aggregation processes can proceed faster compared to aggregates forming under quiescence state. The low shear forces enhance the possibility of mutual interactions among chitosan clusters and bring molecules together in the form of aggregates faster than the Brownian motion does. After exceeding the critical value of shear rate, high shear forces are able to overcome the associative forces merging the chitosan cluster. As a result, chitosan clusters are not only deformed but also break up into smaller aggregates.

The gelation temperature, radius of gyration, and conformation of chitosan molecules were determined based on the oscillatory shear and non-isothermal measurements combined with recording of SALS data. The rheological (crossover point of dynamic moduli and F-L method) and light scattering (Zimm plot) approaches were used to determine values of phase transition temperatures. An analysis of the obtained results allowed one to determine the phase transition area from its sol-like beginning (determined as a crossover point of the moduli) to its gel-like end (determined by light scattering data and F-L approach).

Quantification of the chitosan molecules’ size was achieved by applying the Debye function in order to determine the radius of gyration. The obtained values of the radius of gyration as an indicator of molecule compactness allow one to state that with an increase of chitosan concentration, the molecule size decreases. The above statement indicates that in more concentrated solutions, the chitosan coils are more tangled and smaller but occur in a larger number than in the diluted solutions. Based on the Porod approach related to the fractal dimension, it was found that with an increase of temperature, the level of polymer coil swelling increases as indicated by the decrease in the exponent *n* from the value of 3 (collapsed coil) to 1 (rigid rod).

The conducted research revealed that combined rheological and SALS measurement can provide substantial information about chitosan hydrogel systems. Apart from macroscopic properties, the applied SALS technique yields microscopic properties such as radius of gyration or structural conformation. From the point of view of designing the hydrogel matrices in biomedical applications, such information is relevant to predicting the crosslinked structure and control drug release kinetics.

## Supplementary Materials

The following are available online at http://www.mdpi.com/2073-4360/10/4/431/s1, Figure S1: Schematic presentation of Rheo-SALS system; Figure S2: Schematic presentation of measurement systems: double gap and plate-plate. The top view on sample plane shows different measurement planes.
