# Supplementary material for: The Application of Small-Angle Light Scattering for Rheo-Optical Characterization of Chitosan Colloidal Solutions
Source: Polymers (Basel). 2018 Apr 13;10(4):431. doi: 10.3390/polym10040431 (PMC6415461; doi:10.3390/polym10040431)
Supplement: Supplementary file 1 [file polymers-10-00431-s001.pdf]

Supplementary Materials

The Application of Small Angle Light Scattering for Rheo-Optical Characterization of Chitosan Colloidal Solutions

Piotr Owczarz, Patryk Ziółkowski * and Marek Dziubiński

Department of Chemical Engineering, Lodz University of Technology, Lodz 90-924, Poland; piotr.owczarz@p.lodz.pl (P.O.); marek.dziubinski@p.lodz.pl (M.D.)

* Correspondence: patryk.ziolkowski@edu.p.lodz.pl; Tel.: +48-42-631-3975

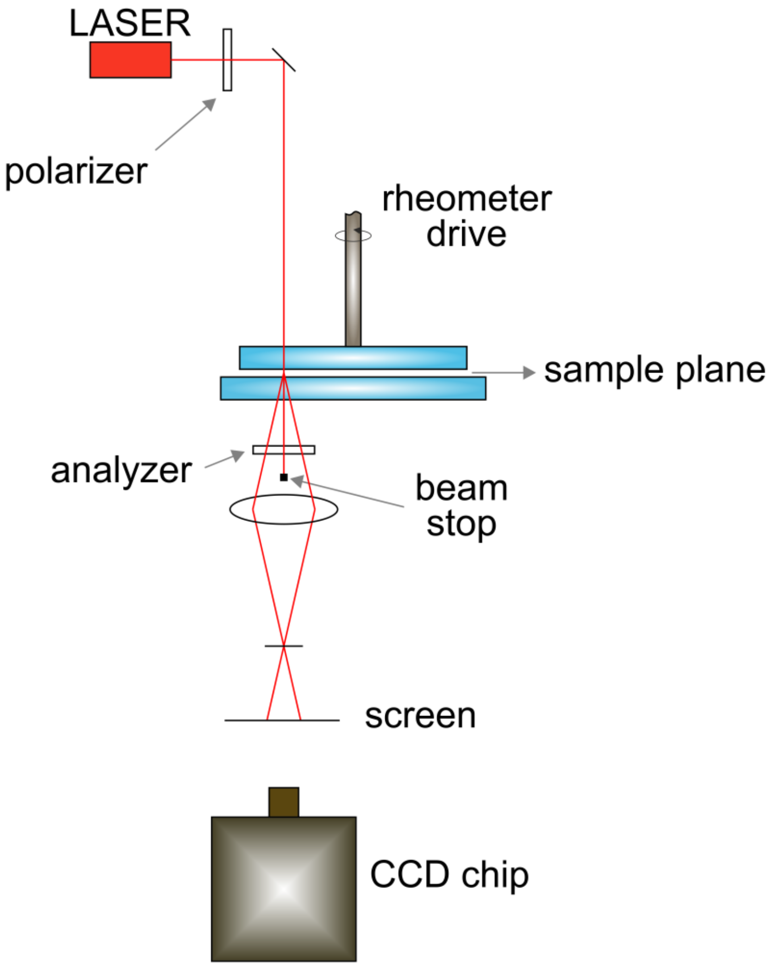

Figure S1. Schematic presentation of Rheo-SALS system.

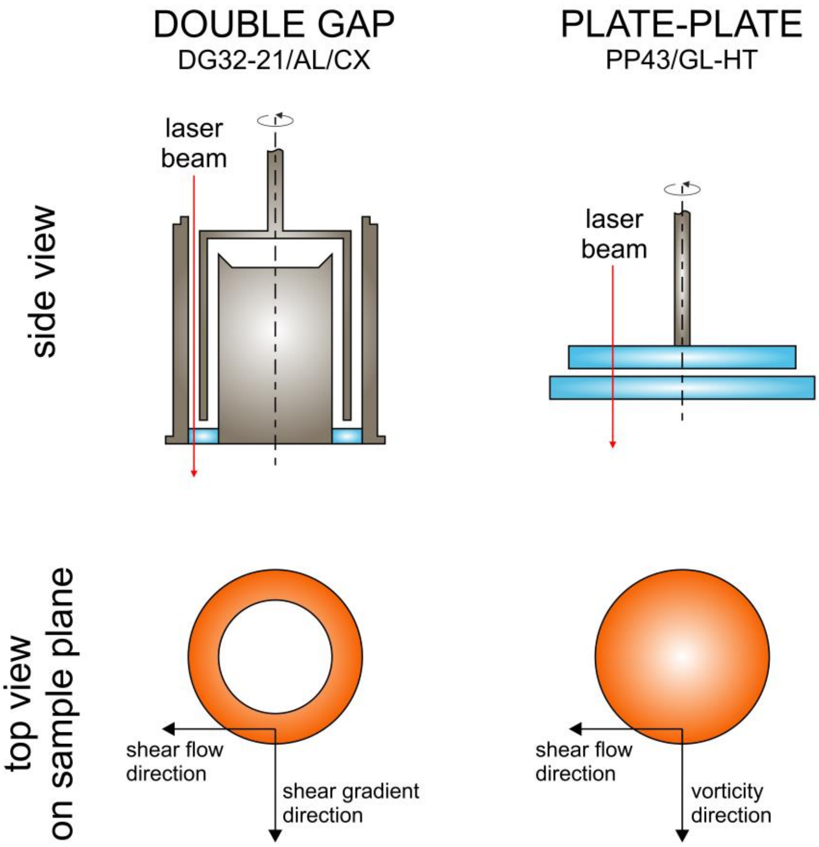

Figure S2. Schematic presentation of measurement systems: double gap and plate-plate. The top view on sample plane shows different measurement planes.
